# Coronary artery disease-associated genetic variants and biomarkers of inflammation

**DOI:** 10.1371/journal.pone.0180365

**Published:** 2017-07-07

**Authors:** Morten Krogh Christiansen, Sanne Bøjet Larsen, Mette Nyegaard, Søs Neergaard-Petersen, Ramzi Ajjan, Morten Würtz, Erik Lerkevang Grove, Anne-Mette Hvas, Henrik Kjærulf Jensen, Steen Dalby Kristensen

**Affiliations:** 1 Department of Cardiology, Aarhus University Hospital, Aarhus, Denmark; 2 Faculty of Health, Institute of Clinical Medicine, Aarhus University, Aarhus, Denmark; 3 Department of Biomedicine, Aarhus University, Aarhus, Denmark; 4 Leeds Institute for Cardiovascular and Metabolic Medicine (LICAMM), University of Leeds, Leeds, United Kingdom; 5 Department of Clinical Biochemistry, Aarhus University Hospital, Aarhus, Denmark; China Medical University, TAIWAN

## Abstract

**Introduction:**

Genetic constitution and inflammation both contribute to development of coronary artery disease (CAD). Several CAD-associated single-nucleotide polymorphisms (SNPs) have recently been identified, but their functions are largely unknown. We investigated the associations between CAD-associated SNPs and five CAD-related inflammatory biomarkers.

**Methods:**

We genotyped 45 CAD-associated SNPs in 701 stable CAD patients in whom levels of high-sensitivity C-reactive protein (hsRCP), interleukin-6, calprotectin, fibrinogen and complement component 3 levels had previously been measured. A genetic risk score was calculated to assess the combined risk associated with all the genetic variants. A multiple linear regression model was used to assess associations between the genetic risk score, single SNPs, and the five inflammatory biomarkers.

**Results:**

The minor allele (G) (CAD risk allele) of rs2075650 (*TOMM40/APOE*) was associated with lower levels of high-sensitivity C-reactive protein (effect per risk allele: -0.37 mg/l [95%CI -0.56 to -0.18 mg/l]). The inflammatory markers tested showed no association with the remaining 44 SNPs or with the genetic risk score.

**Conclusions:**

In stable CAD patients, the risk allele of a common CAD-associated marker at the *TOMM40/APOE* locus was associated with lower hsCRP levels. No other genetic variants or the combined effect of all variants were associated with the five inflammatory biomarkers.

## Introduction

Inflammation is of major importance for the development of coronary artery disease (CAD) [1]. Inflammatory cells and signaling molecules contribute to the disease process by modulating the arterial wall, promoting lipoprotein retention, plaque formation and possibly destabilization [1]. Accordingly, several inflammatory biomarkers have been shown to predict cardiovascular outcome [2–5].

Over the past 10 years, large-scale genome-wide association studies (GWAS) have identified a large number of single-nucleotide polymorphisms (SNPs) associated with CAD [6]. Combined into genetic risk scores (GRS), these SNPs predict adverse cardiovascular events in various populations with and without prior cardiovascular disease [7–10]. Although the majority of loci identified seem to act through induction of atherosclerosis, little is known about the underlying mechanisms.

The majority of CAD-associated SNPs are located in non-coding regions of the genome. Expression quantitative trait loci (eQTL) data indicate that the loci primarily exert their effect through regulation of nearby gene expression, but a large proportion of these genes have not previously been linked to CAD or risk factors for CAD [11,12]. A functional network analysis performed by the CARDIoGRAMplusC4D Consortium (as part of the largest GWAS meta-analysis available at the time of initiation of our study) suggested that SNPs related to the *APOA1*, *IL6R*, *MRAS*, and *PLG* genes may act on CAD development by affecting pathways of acute phase response signaling [11]. However, it remains unknown whether these SNPs are associated with commonly used outcome-associated biomarkers of inflammation such as high-sensitivity C-reactive protein (hs-CRP), interleukin 6 (IL-6), calprotectin, fibrinogen, and complement component 3 (C3) [2–5].

Therefore, our primary aim was to investigate the association between *APOA1*-, *IL6R*-, *MRAS*-, and *PLG*-linked SNPs, and hs-CRP, IL-6, calprotectin, fibrinogen, and C3. Secondarily, we explored the individual associations of other CAD-associated risk SNPs and the effect of all SNPs combined using a genetic risk score (GRS).

## Methods

### Design and study population

This was a cross-sectional study including patients with stable CAD. The entire cohort has previously been described in detail [13]. Briefly, 900 patients were recruited from the Western Denmark Heart Registry between November 2007 and January 2011, and all patients had CAD as verified by coronary angiography. At the time of enrollment, where blood samples were obtained, patients were considered stable (i.e. no cardiovascular events or revascularization procedures within the last 12 months).

From the entire cohort, substudies on inflammatory biomarkers were performed comprising hs-CRP [14], IL-6 [14], calprotectin [14], fibrinogen [15,16], and C3 [15,16]. Patients included in these substudies were younger, more often had diabetes, prior MI and prior coronary revascularization, whereas renal failure and antihypertensive medication was less common, compared with patients not included. In total, one or more inflammatory markers were measured in 713 patients, and DNA was available in 704 patients. All patients provided informed written consent. The project was approved by The Central Denmark Region Committees on Health Research Ethics (record number: 1-10-72-210-15) and by the Danish Data Protection Agency (record number: 1-16-02-400-15).

### Inflammatory marker measurements

Standardized blood sampling was performed in the outpatient clinic between 8 a.m. and 3 p.m. Blood was sampled from the antecubital vein with patients in supine position after 30 minutes of rest using vacuum tubes, a large bore needle (19 G), and a minimum of stasis [13]. Blood for hs-CRP analysis was analysed using the KoneLab 30i (ILS Laboratories Scandinavia, Allerød, Denmark). The measurement range for hs-CRP was 0.2–10.0 mg/l. In 27 patients, hs-CRP values were outside this range (maximum CRP-value of 35.8 mg/l). These patients were excluded in order to avoid bias from patients with subclinical infections which could potentially affect the levels of the inflammatory markers measured. IL-6 analyses were performed using the Cobas^®^ 6000 analyser, E module (Roche, Mannheim, Germany). Serum calprotectin was measured using enzyme-linked immunosorbent assay (ELISA) (MRP 8/14 Calprotectin, Bühlmann, Schönenbuch, Switzerland). Fibrinogen was measured by the clotting method of Clauss using a KC 10^TM^ coagulometer (Henrich Amelung GmbH, Lemgo, Germany). Complement C3 was determined by ELISA according to the manufacturer’s instructions (GenWay Biotech, Inc., San Diego, CA, USA). The coefficient of variance was <5% for both calprotectin and C3 ELISA assays.

### SNP selection and genotyping

A thorough literature review of CAD risk loci was used to select the lead SNPs or relevant proxies of 46 loci genome-wide significantly associated with CAD and/or myocardial infarction (MI) in populations of European ancestry [6]. This included CAD-associated lead SNPs previously linked to the *APOA1*, *IL6R*, *MRAS*, and *PLG* genes by either eQTL data or physical proximity [11].

DNA was obtained from whole blood and direct genotyping was performed on a Fluidigm Biomark HD as previously described [17]. One SNP (rs17114036) failed on all chips and three samples with less than 50% of SNPs successfully genotyped were excluded. Therefore, the final dataset consisted of 45 SNPs in 701 patients. Overall call rate was excellent (31376/31545 = 99.5%) and consistent for all SNPs, except for rs964184 (call rate: 570/701 = 81.3%). All genotypes were successfully called in 559/701 = 79.7% of samples, whereas ≥43 SNPs where successfully called in 697/701 = 99.4% of samples.

### Statistical analysis

Patient characteristics are reported as mean ± standard deviation (SD), median (interquartile range [IQR]) or numbers (percentage). Each SNP was coded as 0, 1, or 2 depending on the number of CAD risk alleles in the patient. Under the assumption of additive genetic effects, a multivariable linear regression model was used to test the association between the individual SNPs and hs-CRP, IL-6, calprotectin, fibrinogen, and C3, respectively. Predefined covariates (age, sex, diabetes, prior MI, current smoking, body mass index [BMI], and renal failure defined as estimated glomerular filtration rate ≤60 ml/min) were simultaneously added to the model. Therefore, the beta coefficient of a SNP corresponds to the adjusted average effect per risk allele on the inflammatory biomarker.

To test the combined effect of all CAD-associated SNPs a weighted GRS was calculated as previously reported [17]. The GRS was calculated as the sum of the number of risk alleles in each individual, weighted by the log of the odds ratio for CAD obtained from the original discovery GWAS papers. In the rare case of a missing genotype, the average of the cohort (a number of 0–2) for that SNP was used to calculate the GRS (in order to avoid a value of zero). For statistical analyses, GRS was standardized meaning that the beta coefficient of the GRS corresponds to the adjusted effect on the inflammatory marker per SD increase in GRS.

In the primary analyses, we considered a conservative Bonferroni-corrected p-value <0.0025 as statistically significant (threshold: p = 0.05 / [4 SNPs × 5 inflammatory biomarkers]). When evaluating the remaining CAD-associated SNPs and the GRS, the level of significance was adjusted accordingly (threshold: p = 0.05 / [46 × 5] = 2.2×10^−4^). All analyses were performed using STATA version 13.1 (StataCorp, 4905 Lakeway Dr, College Station, TX, USA).

## Results

### Patient characteristics

A total of 701 patients were included in data analyses. Patient characteristics and numbers included in each analysis are displayed in Table 1. Mean age was 64 ± 9 years (range: 32–85 years) and 558 (80%) were males. Prior MI, diabetes and renal failure were present in 627 (89%), 218 (31%), and 102 (15%) of the patients, respectively.

**Table 1:** Patient characteristics.

	hs-CRP^a^ (n = 484)	IL-6^b^ (n = 563)	Calprotectin^b^ (n = 543)	Fibrinogen^c^ (n = 700)	C3^c^ (n = 698)
Age	64 ± 9	64 ± 9	64 ± 9	65 ± 9	65 ± 9
Male sex	386 (80)	446 (79)	430 (79)	558 (80)	557 (80)
Prior MI	442 (91)	518 (92)	499 (92)	626 (89)	624 (89)
Prior PCI/CABG	471 (97)	544 (97)	524 (97)	672 (96)	670 (96)
Prior Stroke	25 (5)	25 (4)	25 (5)	37 (5)	37 (5)
Diabetes	107 (22)	140 (25)	135 (25)	218 (31)	218 (31)
Renal failure^d^	66 (14)	77 (14)	76 (14)	102 (15)	102 (15)
Antihypertensive treatment	437 (90)	509 (91)	493 (91)	636 (91)	635 (91)
Statin treatment	448 (93)	519 (92)	501 (92)	635 (91)	634 (91)
Current smoking	98 (20)	117 (21)	113 (21)	150 (21)	150 (22)
Systolic BP (mmHg)	142 ± 20	142 ± 21	142 ± 20	142 ± 21	142 ± 21
Diastolic BP (mmHg)	83 ± 11	83 ± 11	83 ± 11	83 ± 11	83 ± 11
Body mass index (kg/m2)	27.6 ± 4.3	27.7 ± 4.4	27.8 ± 4.4	27.9 ± 4.3	27.9 ± 4.4
Creatinine (mM)	81 (71–93.5)	81 (71–93)	81 (71–93)	81 (71–93.5)	81 (71–94)

Data are presented as mean ± SD, median (IQR), or n (%).Abbreviations: BMI, body mass index; BP, blood pressure; CABG, coronary artery bypass graft surgery; MI, myocardial infarction; PCI, percutaneous coronary intervention.

^a^ Data on prior stroke, antihypertensive treatment, statin treatment, BP, and BMI were missing in 3, 1, 1, 25, and 2 individuals, respectively.

^b^ Data on prior stroke, antihypertensive treatment, statin treatment, current smoking, BP, and BMI were missing in 4, 1, 1, 1, 28, and 2 individuals, respectively.

^c^ Data on prior stroke, antihypertensive treatment, statin treatment, current smoking, BP, and BMI were missing in 6, 2, 3, 1, 30, and 2 individuals, respectively.

^d^ Estimated glomerular filtration rate ≤60 ml/min.

### Presumed inflammation-related SNPs and inflammatory proteins

The association between presumed inflammation-related SNPs and hs-CRP, IL-6, calprotectin, fibrinogen, and C3 is presented in Table 2. A weak association was observed between rs4845625 (*IL6R*) and C3 (mean adjusted effect per risk allele: 0.03 (95% CI 0.00–0.06) mg/ml, p = 0.04), but it did not meet the Bonferroni-corrected threshold of significance. Neither rs4252120 (*PLG*) rs964184 (*APOA1*), nor rs9818870 (*MRAS*) significantly affected the inflammatory markers measured.

**Table 2:** Associations between presumed inflammation-related SNPs and inflammatory proteins.

Locus	SNP	Nearby genes	Call rate (%)	RAF	hs-CRP Beta (95% CI)	p	Interleukin-6 Beta (95% CI)	p	Calprotectin Beta (95% CI)	p	Fibrinogen Beta (95% CI)	p	Complement C3 Beta (95% CI)	p
6q26	rs4252120	*PLG*	100	0.70	-0.01 (-0.16–0.15)	0.92	-0.56 (-1.13–0.02)	0.061	0.06 (-0.01–0.14)	0.10	0.02 (-0.09–0.14)	0.69	-0.01 (-0.04–0.03)	0.72
1q21.3	rs4845625	*IL6R*	99.6	0.44	0.10 (-0.04–0.25)	0.17	0.14 (-0.40–0.68)	0.61	0.00 (-0.07–0.07)	0.98	0.09 (-0.02–0.19)	0.12	0.03 (0.00–0.06)	**0.042**
11q23.3	rs964184	*APOA1*	81.3	0.15	0.15 (-0.05–0.36)	0.14	0.05 (-0.71–0.82)	0.89	0.04 (-0.06–0.14)	0.45	-0.04 (-0.15–0.07)	0.49	0.02 (-0.03–0.06)	0.52
3q22.3	rs9818870	*MRAS*	99.9	0.18	-0.02 (-0.20–0.15)	0.79	-0.24 (-0.91–0.44)	0.49	-0.02 (-0.11–0.06)	0.59	-0.04 (-0.17–0.10)	0.59	-0.01 (-0.05–0.03)	0.63

Beta is the adjusted mean difference measured per risk allele. Bold indicate that the SNP meets a nominal threshold of significance of p<0.05. Abbreviations: hs-CRP, high-sensitivity C-reactive protein; RAF, risk allele frequency; SNP, single nucleotide polymorphism.

### Remaining CAD-related SNPs and inflammatory proteins

**Fig 1:**
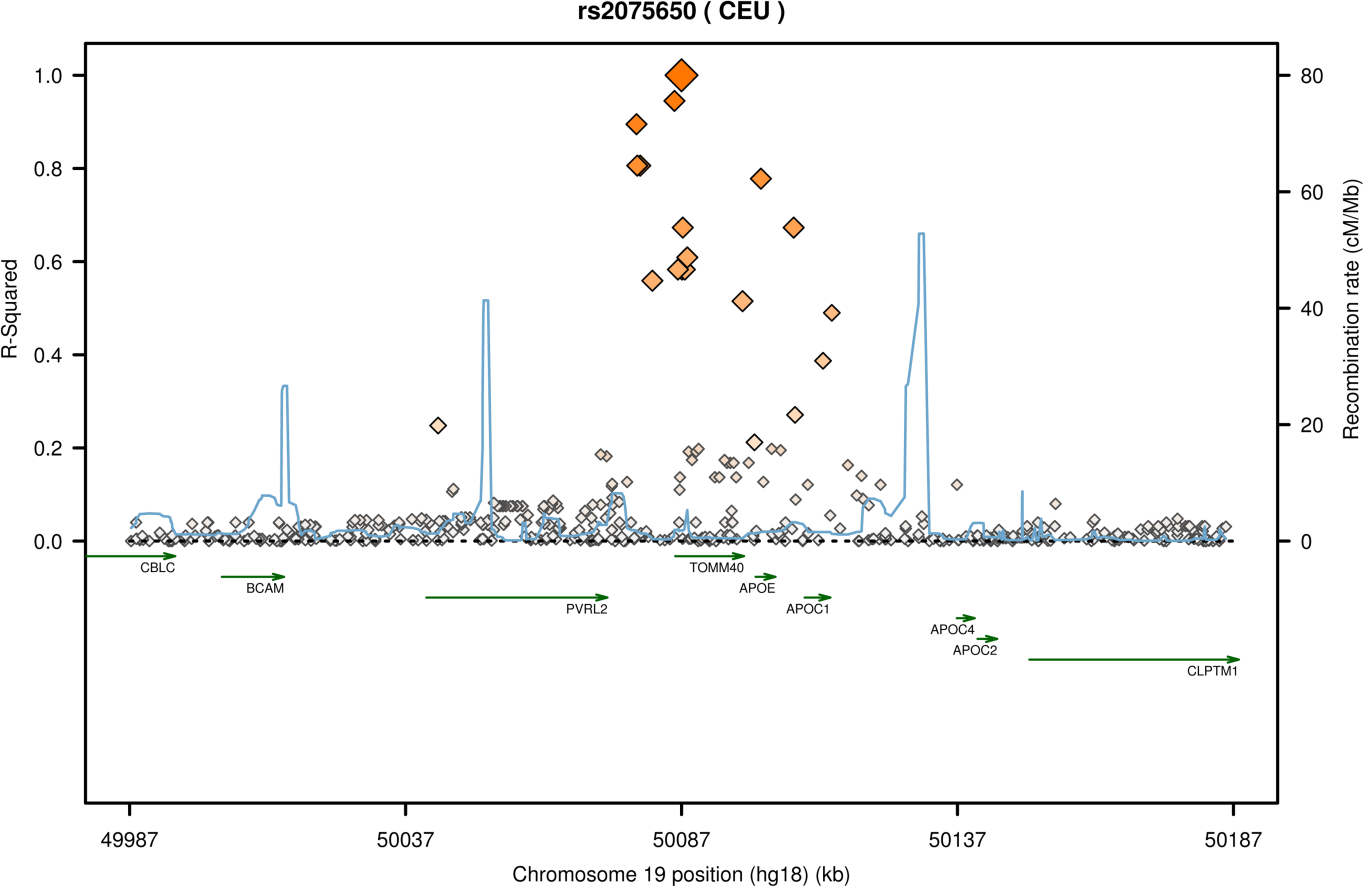
Recombination rate and linkage disequilibrium at the rs2075650 locus in the CEU population. Generated using SNAP (http://archive.broadinstitute.org/mpg/snap/ldplot.php) [18].

**Fig 2:**
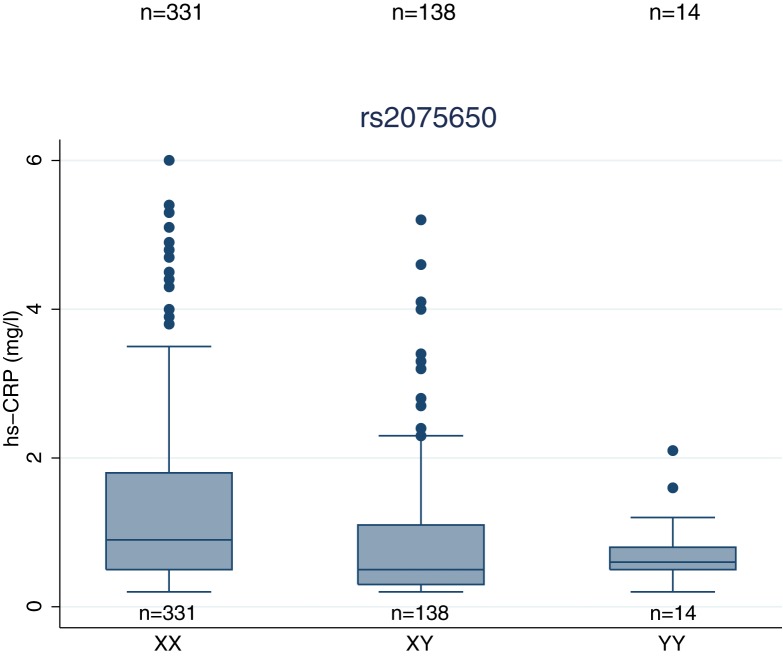
Distribution of hs-CRP levels stratified by the genotypes of rs2075650. Boxes and whiskers indicate quartiles and adjacent values. Values outside the range of adjacent values are plotted as outliers. Abbreviations: hs-CRP, high sensitivity C-reactive protein.

The evaluation of the remaining CAD-related SNPs is presented in Table 3. Of these, a nominally significant association was observed for rs1561198 (*VAMP5*/*VAMP8*) with IL-6 and calprotectin; rs17609940 (*ANKS1A*) with C3; rs2075650 (*TOMM40*, *APOE*) with hs-CRP and IL-6; rs264 (*LPL*) with C3; and finally rs599839 (*SORT1*) with fibrinogen. Only the association between rs2075650 and hs-CRP met the Bonferroni-corrected threshold of significance. The rs2075650 locus is displayed in Fig 1. Further analysis showed that mean level of hs-CRP in wildtype homozygous (A/A), heterozygous (A/G), and risk allele homozygous (G/G) patients were 1.38 mg/l (95% CI 1.25–1.52 mg/l), 0.96 mg/l (95% CI 0.79–1.13 mg/l), and 0.81 mg/l (95% CI 0.52–1.10 mg/l), respectively (Fig 2), demonstrating a gene-related dose-response effect.

**Table 3:** Associations between remaining CAD-related SNPs and inflammatory proteins.

Locus	SNP	Nearby genes	Call rate (%)	RAF	hs-CRP Beta (95% CI)	p	IL-6 Beta (95% CI)	p	Calprotectin Beta (95% CI)	p	Fibrinogen Beta (95% CI)	p	C3 Beta (95% CI)	p
6p21.2	rs10947789	*KCNK5*	100	0.80	0.10 (-0.09–0.29)	0.29	-0.23 (-0.92–0.46)	0.51	0.00 (-0.09–0.09)	0.95	0.01 (-0.12–0.15)	0.85	0.01 (-0.03–0.04)	0.68
7q22	rs10953541	*BCAP29*	100	0.76	-0.06 (-0.22–0.11)	0.50	0.04 (-0.58–0.66)	0.90	-0.02 (-0.10–0.06)	0.64	-0.03 (-0.15–0.09)	0.62	0.01 (-0.02–0.04)	0.61
1p32.3	rs11206510	*PCSK9*	100	0.81	-0.06 (-0.24–0.12)	0.50	0.25 (-0.40–0.91)	0.45	0.05 (-0.04–0.13)	0.27	0.01 (-0.12–0.14)	0.85	0.01 (-0.02–0.05)	0.53
19p13.2	rs1122608	*LDLR*	100	0.78	-0.05 (-0.22–0.12)	0.56	0.14 (-0.50–0.79)	0.66	0.03 (-0.05–0.11)	0.49	0.06 (-0.07–0.19)	0.37	0.02 (-0.02–0.06)	0.27
7q32.2	rs11556924	*ZC3HC1*	100	0.63	-0.04 (-0.19–0.12)	0.65	0.23 (-0.35–0.80)	0.44	0.00 (-0.08–0.07)	0.99	-0.03 (-0.14–0.08)	0.62	0.00 (-0.03–0.03)	0.99
6q23.2	rs12190287	*TCF21*	100	0.65	-0.13 (-0.28–0.03)	0.10	0.00 (-0.58–0.57)	0.99	0.02 (-0.05–0.10)	0.52	-0.02 (-0.14–0.09)	0.70	0.01 (-0.02–0.04)	0.59
10q24.3	rs12413409	*CYP17A1*	100	0.90	-0.09 (-0.33–0.14)	0.44	0.35 (-0.54–1.23)	0.44	-0.04 (-0.16–0.07)	0.46	-0.08 (-0.26–0.10)	0.38	0.01 (-0.03–0.06)	0.55
6p24.1	rs12526453	*PHACTR1*	99.9	0.69	-0.05 (-0.20–0.10)	0.53	-0.12 (-0.69–0.45)	0.67	0.04 (-0.04–0.11)	0.33	-0.01 (-0.12–0.10)	0.87	0.02 (-0.01–0.05)	0.29
17p11.2	rs12936587	*RASD1*. *SMCR3*. *PEMT*	100	0.54	0.01 (-0.14–0.17)	0.85	0.10 (-0.46–0.66)	0.73	-0.02 (-0.09–0.05)	0.65	-0.04 (-0.15–0.07)	0.49	0.01 (-0.02–0.04)	0.62
9p21.3	rs1333049	*CDKN2BAS (ANRIL)*	99.9	0.51	-0.06 (-0.21–0.08)	0.40	0.15 (-0.38–0.69)	0.58	0.01 (-0.06–0.08)	0.83	0.07 (-0.04–0.17)	0.21	0.00 (-0.03–0.03)	0.92
10q23	rs1412444	*LIPA*	99.9	0.34	0.00 (-0.15–0.14)	0.98	-0.25 (-0.79–0.30)	0.38	0.00 (-0.07–0.07)	0.92	0.01 (-0.10–0.12)	0.89	0.02 (-0.01–0.05)	0.22
2p11.2	rs1561198	*VAMP5*. *VAMP8*	100	0.48	-0.05 (-0.19–0.09)	0.47	-0.58 (-1.10–-0.05)	**0.033**	-0.09 (-0.15–-0.02)	**0.014**	0.00 (-0.11–0.10)	1.00	-0.02 (-0.05–0.01)	0.12
1q41	rs17465637	*MIA3*	99.9	0.77	-0.04 (-0.21–0.13)	0.63	-0.18 (-0.81–0.44)	0.56	0.01 (-0.07–0.09)	0.82	-0.05 (-0.18–0.07)	0.40	0.00 (-0.04–0.03)	0.89
6p21.31	rs17609940	*ANKS1A*	99.7	0.78	0.06 (-0.12–0.23)	0.53	0.34 (-0.30–0.97)	0.30	-0.05 (-0.13–0.03)	0.21	0.02 (-0.11–0.15)	0.77	-0.05 (-0.08–-0.02)	**0.0044**
4q31.22	rs1878406*	*EDNRA*	100	0.15	0.20 (0.00–0.40)	**0.047**	-0.11 (-0.84–0.63)	0.77	0.03 (-0.06–0.13)	0.48	0.01 (-0.14–0.15)	0.94	-0.02 (-0.06–0.02)	0.29
7p21.1	rs2023938	*HDAC9*	100	0.10	0.22 (-0.02–0.47)	0.072	0.56 (-0.36–1.49)	0.23	-0.06 (-0.18–0.06)	0.31	0.00 (-0.17–0.18)	0.96	0.00 (-0.05–0.05)	0.92
19q13	rs2075650	*TOMM40*, *APOE*	99.9	0.17	-0.37 (-0.56–-0.18)	**1.4×10**^**−4**^	-0.73 (-1.45–-0.02)	**0.046**	-0.06 (-0.15–0.03)	0.19	0.05 (-0.10–0.19)	0.54	0.00 (-0.04–0.04)	0.94
17p13.3	rs216172	*SMG6*	99.7	0.38	0.00 (-0.15–0.15)	0.99	-0.02 (-0.44–0.40)	0.93	-0.04 (-0.11–0.03)	0.26	0.06 (-0.05–0.17)	0.31	-0.03 (-0.06–0.00)	0.061
2q22.3	rs2252641	*ZEB2*	100	0.48	-0.11 (-0.26–0.04)	0.14	0.30 (-0.26–0.85)	0.30	0.01 (-0.06–0.08)	0.75	0.05 (-0.06–0.16)	0.38	-0.02 (-0.05–0.01)	0.20
10p11.23	rs2505083	*KIAA1462*	100	0.43	0.12 (-0.03–0.27)	0.13	0.11 (-0.44–0.67)	0.69	0.00 (-0.08–0.07)	0.89	0.10 (-0.01–0.21)	0.072	-0.02 (-0.05–0.01)	0.27
8p21.3	rs264	*LPL*	99.6	0.86	0.16 (-0.05–0.38)	0.13	0.40 (-0.20–1.00)	0.19	-0.03 (-0.13–0.07)	0.54	-0.05 (-0.20–0.10)	0.53	0.04 (0.00–0.08)	**0.049**
5q31.1	rs273909	*SLC22A4*	100	0.12	-0.14 (-0.37–0.09)	0.24	-0.26 (-1.13–0.61)	0.56	-0.01 (-0.12–0.10)	0.86	-0.16 (-0.33–0.01)	0.063	0.00 (-0.05–0.04)	0.93
14q32.2	rs2895811	*HHIPL1*	99.9	0.47	-0.02 (-0.17–0.12)	0.76	-0.32 (-0.73–0.10)	0.13	-0.01 (-0.08–0.06)	0.79	0.03 (-0.08–0.14)	0.55	0.01 (-0.02–0.04)	0.69
8q24.13	rs2954029	*TRIB1*	100	0.52	-0.14 (-0.29–0.01)	0.061	-0.40 (-0.95–0.14)	0.15	-0.05 (-0.12–0.02)	0.18	-0.04 (-0.15–0.07)	0.50	-0.01 (-0.04–0.02)	0.49
12q24.12	rs3184504	*SH2B3*	99.0	0.56	0.01 (-0.13–0.16)	0.89	-0.25 (-0.78–0.28)	0.35	-0.05 (-0.12–0.02)	0.16	0.00 (-0.10–0.11)	0.93	0.00 (-0.03–0.03)	0.96
6q25.3	rs3798220	*LPA*	100	0.02	0.02 (-0.48–0.52)	0.94	-0.81 (-2.81–1.20)	0.43	-0.14 (-0.39–0.11)	0.27	-0.02 (-0.41–0.37)	0.92	-0.08 (-0.18–0.03)	0.16
15q25.1	rs3825807	*ADAMTS7*	99.7	0.60	0.03 (-0.11–0.18)	0.67	0.24 (-0.31–0.78)	0.39	-0.01 (-0.08–0.06)	0.79	0.05 (-0.06–0.16)	0.35	0.01 (-0.02–0.04)	0.52
6p21.33	rs3869109	*HLA-C*. *HLA-B*	99.7	0.59	-0.09 (-0.23–0.05)	0.22	0.18 (-0.35–0.71)	0.50	0.06 (-0.01–0.12)	0.10	-0.10 (-0.21–0.00)	0.062	0.02 (-0.01–0.04)	0.29
17q21.32	rs46522	*UBE2Z*	100	0.58	-0.10 (-0.25–0.05)	0.19	-0.04 (-0.59–0.51)	0.89	0.06 (-0.01–0.13)	0.12	0.07 (-0.04–0.18)	0.23	0.00 (-0.03–0.02)	0.75
13q34	rs4773144	*COL4A1*	99.7	0.42	0.01 (-0.14–0.16)	0.89	-0.25 (-0.82–0.32)	0.38	-0.01 (-0.08–0.07)	0.89	0.01 (-0.10–0.12)	0.90	-0.03 (-0.06–0.00)	0.083
9q34	rs495828	*AB0*	99.7	0.23	0.09 (-0.08–0.26)	0.29	0.40 (-0.23–1.03)	0.21	0.01 (-0.07–0.09)	0.87	0.00 (-0.13–0.12)	0.98	0.01 (-0.02–0.05)	0.46
10q11.1	rs501120	*CXCL12*	100	0.88	-0.08 (-0.30–0.14)	0.48	-0.25 (-1.07–0.57)	0.56	0.10 (-0.01–0.20)	0.076	-0.04 (-0.19–0.12)	0.66	0.02 (-0.02–0.07)	0.28
2p24.1	rs515135	*APOB*	99.9	0.83	0.10 (-0.09–0.28)	0.31	0.69 (-0.01–1.38)	0.053	0.05 (-0.04–0.14)	0.26	-0.01 (-0.15–0.13)	0.88	-0.01 (-0.05–0.03)	0.54
1p13	rs599839	*SORT1*	100	0.78	0.15 (-0.03–0.33)	0.10	0.21 (-0.45–0.88)	0.53	0.06 (-0.02–0.15)	0.16	0.14 (0.01–0.28)	**0.034**	0.01 (-0.02–0.05)	0.54
2p21	rs6544713	*ABCG8*	100	0.30	-0.04 (-0.20–0.12)	0.62	0.06 (-0.53–0.66)	0.83	-0.05 (-0.12–0.03)	0.22	-0.02 (-0.13–0.10)	0.80	-0.01 (-0.04–0.02)	0.54
2q33.1	rs6725887	*WDR12*	100	0.17	-0.14 (-0.33–0.05)	0.15	0.01 (-0.71–0.74)	0.97	0.02 (-0.07–0.11)	0.68	-0.08 (-0.22–0.06)	0.29	0.01 (-0.03–0.05)	0.54
4q32.1	rs7692387	*GUCY1A3*	100	0.83	-0.13 (-0.33–0.07)	0.22	0.09 (-0.66–0.85)	0.80	0.00 (-0.10–0.10)	0.98	-0.05 (-0.19–0.10)	0.51	-0.02 (-0.06–0.02)	0.39
15q26.1	rs8039305	*FURIN*	99.7	0.51	-0.01 (0.16–0.13)	0.85	0.27 (-0.28–0.82)	0.33	-0.01 (-0.08–0.06)	0.87	-0.03 (-0.14–0.08)	0.56	0.03 (0.00–0.06)	0.068
13q12.3	rs9319428	*FLT1*	99.7	0.33	0.00 (-0.15–0.16)	0.96	0.24 (-0.33–0.82)	0.41	-0.03 (-0.11–0.04)	0.37	0.00 (-0.11–0.12)	0.95	-0.01 (-0.04–0.02)	0.66
11q22.3	rs974819	*PDGFD*	100	0.27	-0.04 (-0.20–0.12)	0.62	0.18 (-0.42–0.79)	0.55	0.03 (-0.05–0.11)	0.45	0.02 (-0.10–0.14)	0.75	-0.02 (-0.05–0.01)	0.24
21q22.1	rs9982601	*MRPS6*	99.9	0.14	0.14 (-0.07–0.35)	0.18	0.22 (-0.57–1.01)	0.59	0.06 (-0.04–0.16)	0.24	-0.02 (-0.17–0.14)	0.84	0.03 (-0.01–0.07)	0.16

Bold values indicate that the SNP meets a nominal threshold of significance of p<0.05.

*rs1878406 was genotyped as a C/T SNP.

Abbreviations: hs-CRP, high-sensitivity C-reactive protein; RAF, risk allele frequency; SNP, single nucleotide polymorphism.

### GRS and inflammatory proteins

**Fig 3:**
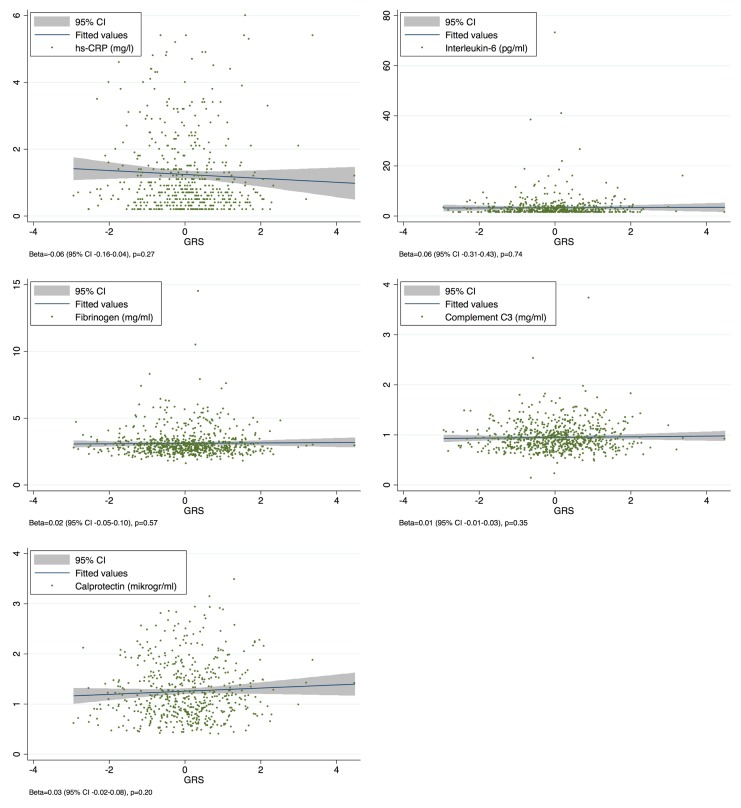
Scatterplots of the association between inflammatory markers and the standardized GRS. Abbreviations: GRS, genetic risk score; hs-CRP, high-sensitivity C-reactive protein.

We found no associations between the GRS and hs-CRP, IL-6, calprotectin, fibrinogen, or C3 with results detailed in Fig 3.

## Discussion

In the present study of patients with established CAD, we investigated the association between 45 lead SNPs from loci associated with CAD and five common biochemical markers of inflammation. The main findings were; 1) for SNP rs2075650 in the *TOMM40*, *APOE* locus, the established CAD-risk allele was significantly associated with lower hs-CRP levels, 2) No other CAD-related SNPs were associated with the inflammatory marker levels, either measured as individual SNPs or when combined into a GRS.

Previous GWASs have demonstrated a robust association between the rs2075650 G-allele and an increased risk of CAD [11]. In a recent study based on the present cohort, subanalysis also confirmed an association between rs2075650 and recurrent CAD events showing that an increase in the number of CAD risk alleles was associated with a hazard ratio of 1.40 (95% CI 1.00–1.97) of the primary endpoint composed of cardiovascular death, myocardial infarction and stable coronary revascularization [19]. Considering the well-established relationship between increasing levels of hs-CRP and adverse cardiovascular outcome [20], it may be surprising that the CAD risk allele of rs2075650 was associated with lower levels of hs-CRP in our sample. However, our findings are consistent with results from several previous large population-based cohort studies. In these studies, the same inverse relationship between the CAD risk allele and lower levels of hs-CRP has also been observed in European Americans [21], Australian twin families [22], Asians [23], and Hispanics [21] but not Afro-Americans [21]. Our work extends these findings by demonstrating an association in patients with established CAD. The mechanistic explanation for this inverse association is currently unknown. The marker rs2075650 is located in the *TOMM40* gene, just upstream of *APOE*, and *APOC1*. The CAD risk allele (G) has been associated with a range of other phenotypes including reduced longevity [24], reduced BMI [25], increased low-density lipoprotein cholesterol (LDL-C) [22,26], and an increased risk of Alzheimer’s disease [27]. Because of the relatively strong linkage disequilibrium in the *TOMM40*/*APOE* locus, it has been suggested that the G-allele at rs2075650 is in fact tagging causal variation in the *APOE* gene. The *APOE* encodes the apolipoprotein E with three different isoforms (ε2, ε3, and ε4 defined by the combination of rs7412 and rs429358. Northwestern European ancestry (CEU): r^2^ = 0.02 and r^2^ = 0.20 with rs2075650, respectively), of which the ε4 isoform has long been known to associate with LDL-C, Alzheimer’s disease, and hs-CRP [28,29]. However, recent data suggest that the *TOMM40*/*APOE* locus is genetically complex [30], and therefore it is plausible that the G-allele is tagging different underlying causal variants with different effects on CAD risk and hs-CRP, a concept supported by Middleberg et al [22]. This would also be in line with the current understanding that hs-CRP is not causally related to cardiovascular risk [31].

Some previous GWASs have explored the association between CAD-associated risk variants and common inflammatory markers, of which the *IL6R* locus has been associated with several. In studies of hs-CRP, the *IL6R* locus (rs4129267) was consistently, though moderately, associated with hs-CRP levels (CEU: r^2^ = 0.54 with rs4845625) [32–34]. Furthermore, *IL6R* (rs4129267) has been associated with plasma levels of fibrinogen and IL-6 [35–37]. Although we observed a nominal association between the *IL6R* locus and C3, our study does not support a significant effect of *IL6R* on the inflammatory response. Other CAD-associated loci have also emerged in GWASs of inflammatory markers. A large study from the CHARGE (Cohorts for Heart and Aging Research in Genetic Epidemiology) consortium demonstrated a significant association between fibrinogen and variants near *LIPA* (rs2250644) and *SH2B3* (rs7310615) [36]. Although these variants are in perfect linkage disequilibrium with the SNPs genotyped in our study (CEU: r^2^ = 1.00 for both), we did not find evidence of such association. Other GWASs have also demonstrated weak associations between variants at the *AB0* locus (rs657152 and rs8176704; CEU: r^2^ = 0.46 and r^2^ = 0.02 with rs495828) and IL-6 [34,37], and a Chinese GWAS of C3 found an association with rs11575839 close to *HLA-C* (CEU: r^2^ = 0.02 with rs3869109) [38]. We were not able to confirm any of these associations. Importantly, our study was not powered to detect very small effect sizes. However, it is striking that none of our estimates indicated even a trend towards such relationships. Several explanations for these inconsistencies may exist. Some of the SNPs tagged in prior GWASs display different allele frequencies compared with the CAD-associated lead SNPs genotyped in our study and slightly different ancestral origins may possibly play a role as well. However, another important explanation may relate to the fact that we included patients with established CAD in contrast to prior studies performed in population-based cohorts without known cardiovascular disease. Patients with CAD have an increased inflammatory response compared with healthy subjects [2], either as the cause or as a consequence of CAD. Therefore, causal genetic variants might not associate with the levels of inflammatory biomarkers in cohorts where all patients are affected by CAD, although such an association may be evident in community-based populations, where some patients likely have subclinical CAD. In this context, it is important to note that we included stable CAD patients in our study. Ninety percent had previous MI occurring at least 12 months prior to inclusion, thus making it less likely that prior MIs influenced the levels of inflammatory biomarkers.

Calprotectin is suggested a new biomarker of CAD [39–41]. The expression of calprotectin has been found at the site of plaque rupture and in macrophages of atherosclerotic plaques and is considered an inflammatory marker of plaque instability [40,42]. To our knowledge, the present study is the first to explore the association between calprotectin and CAD-associated risk variants. Although none of the CAD-associated variants significantly affected calprotectin levels, a trend was observed for rs1561198. This SNP is located between the *VAMP5* and *VAMP8* genes, whose products are involved in different aspects of vesicle trafficking including cytokine release and phagocytosis [43]. Hence, a link between this locus and calprotectin levels may plausibly exist. However, further studies with larger number of individuals are needed to confirm this hypothesis.

Our study has limitations. Because of the number of statistical tests performed, we applied a conservative Bonferroni-corrected threshold of significance to reduce the risk of type I errors. This, together with the moderate sample size in the context of common complex diseases, reduces the power to detect small effect sizes, in particular for SNPs with low minor allele frequencies and for hs-CRP, IL-6, and calprotectin, which were not assessed in all patients. Therefore, our study should be considered as exploratory. We did not assess the presence of other inflammatory conditions, which may also affect the levels of inflammatory biomarkers. In case of bias, this would likely lead the associations towards the null, since no strong association between the genotyped SNPs and any such conditions has been reported. Moreover, we performed the statistical analyses assuming additive genetic effects of the risk alleles. Although this assumption may be reasonable for most of the genetic loci investigated, some might better fit a recessive model [44], which would affect the power of our analyses.

## Conclusion

In the present study, a common CAD-associated variant at the *TOMM40*/*APOE* locus (rs2075650) was significantly associated with lower levels of hs-CRP in patients with stable CAD. Future studies using deep sequencing of the *TOMM40*/*APOE* locus in large clinical samples are warranted to determine if rs2075650 is truly causing opposite allelic effects on CAD and hs-CRP, or if the opposite association is explained by underlying linkage disequilibrium with several hidden functional variants of which some affect the development of CAD independent of hs-CRP. None of the remaining variants, both assessed independently or combined as a GRS, were associated with hs-CRP, IL-6, calprotectin, fibrinogen, or C3. Our findings may suggest that the effect of these CAD-loci on CAD development does not act through pathways significantly affecting these commonly used inflammatory biomarkers.

## Supporting information

S1 File Competing interests statement. (PDF)

S2 File Dataset file. (DTA)
