# Supplementary material for: Coronary artery disease-associated genetic variants and biomarkers of inflammation
Source: PLoS One. 2017 Jul 7;12(7):e0180365. doi: 10.1371/journal.pone.0180365 (PMC5501546; doi:10.1371/journal.pone.0180365)
Supplement: S1 File — (PDF) [file pone.0180365.s001.pdf]

Competing Interests Statement

Regarding the manuscript entitled: "Coronary artery disease-associated genetic variants and biomarkers of inflammation"

HKJ has received an unrestricted research grant from Pfizer, which was partly used to finance a minor part of the genetic analyses.

This does not alter our adherence to PLOS ONE policies on sharing data and materials.

All other authors report no competing interests.

On behalf of all authors,

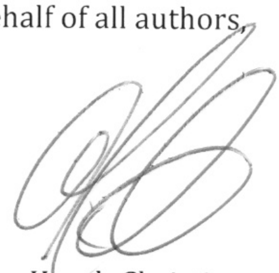

Morten Krogh Christiansen (*corresponding author*)
MD, PhD
Aarhus University Hospital
Department of Cardiology
Palle Juul-Jensens Boulevard 99
DK-8200 Aarhus
Denmark
